# Assessing service use for mental health by Indigenous populations in Australia, Canada, New Zealand and the United States of America: a rapid review of population surveys

**DOI:** 10.1186/s12961-017-0233-5

**Published:** 2017-08-04

**Authors:** Cecily McIntyre, Meredith G. Harris, Amanda J. Baxter, Stuart Leske, Sandra Diminic, Joseph P. Gone, Ernest Hunter, Harvey Whiteford

**Affiliations:** 1 School of Public Health, The University of Queensland, Herston, Queensland Australia; 2 Yale University, New Haven, CT United States of America; 3 Queensland Centre for Mental Health Research, The Park – Centre for Mental Health, Wacol, Queensland Australia; 4 Remote Area Mental Health Service, Queensland Health, Cairns, Queensland Australia; 5 Department of Psychology, University of Michigan, Ann Arbor, MI United States of America

**Keywords:** Indigenous, Mental health services, Mental disorders, Surveys and questionnaires

## Abstract

**Background:**

Indigenous people in Australia, Canada, New Zealand and the United States of America experience disproportionately poor mental health compared to their non-Indigenous counterparts. To optimally allocate resources, health planners require information about the services Indigenous people use for mental health, their unmet treatment needs and the barriers to care. We reviewed population surveys of Indigenous people to determine whether the information needed to guide service development is being collected.

**Methods:**

We sought national- or state-level epidemiological surveys of Indigenous populations conducted in each of the four selected countries since 1990 that asked about service use for mental health. Surveys were identified from literature reviews and web searches. We developed a framework for categorising the content of each survey. Using this framework, we compared the service use content of the surveys of Indigenous people to each other and to general population mental health surveys. We focused on identifying gaps in information coverage and topics that may require Indigenous-specific questions or response options.

**Results:**

Nine surveys met our inclusion criteria. More than half of these included questions about health professionals consulted, barriers to care, perceived need for care, medications taken, number, duration, location and payment of health professional visits or use of support services or self-management. Less than half included questions about interventions received, hospital admissions or treatment dropout. Indigenous-specific content was most common in questions regarding use of support services or self-management, types of health professionals consulted, barriers to care and interventions received.

**Conclusions:**

Epidemiological surveys measuring service use for mental health among Indigenous populations have been less comprehensive and less standardised than surveys of the general population, despite having assessed similar content. To better understand the gaps in mental health service systems for Indigenous people, systematically-collected subjective and objective indicators of the quality of care being delivered are needed.

**Electronic supplementary material:**

The online version of this article (doi:10.1186/s12961-017-0233-5) contains supplementary material, which is available to authorized users.

## Background

There exists a strong consensus that Indigenous populations of Australia, Canada, New Zealand and the United States of America experience mental health inequities relative to the non-Indigenous population. To address this disparity and optimally allocate resources, health planners require population-level information about how many Indigenous people are using mental health services, the types of interventions they receive, the extent to which these interventions align with best available evidence, their unmet needs for care and the barriers to care [1]. A content review of existing service use modules in Indigenous surveys is required to determine whether the information needed to guide service development is being collected and to inform future survey design.

While wary of the danger of pathologising Indigenous communities [2], some epidemiological surveys and anecdotal evidence suggests that Indigenous populations in these four countries experience a higher disability and mortality burden from mental disorders and substance abuse than their non-Indigenous counterparts [3–7]. However, findings are not consistent across studies; this may reflect methodological differences as well as within and between-group diversity across Indigenous populations. For instance, an epidemiological study in the United States, which looked at mental disorders among the Southwest and Northern Plains Native Americans, found that neither group experienced a higher overall burden of mental disorders as compared to the general United States population, yet both experienced disparities (to different degrees) in particular disorders, such as lifetime alcohol dependence and lifetime post-traumatic stress disorder [8]. The disparities exist despite these countries having well-established mental health service systems that offer a range of primary and specialised mental health services delivered by private, public and non-government providers. In addition to mainstream mental health services, each of these countries funds public programmes to deliver mental health services specifically for Indigenous people such as the Indian Health Service in the United States [9] and the National Aboriginal Community Controlled Health Organisation in Australia [10]. Table 1 provides a summary of the health service systems in the four countries.

**Table 1:** Comparison of mental health service systems in Australia, New Zealand, Canada and the United States

	Australia	Canada	New Zealand	United States
Health service system and private insurance role	Universal public medical insurance programme (Medicare); regionally administered public hospital funding ~47.3% buy complementary and supplementary coverage	Universal public medical insurance programme that plans and funds (mainly private) provision, administered separately by each province/territory ~67% buy complementary coverage for non-covered benefits	National healthcare system; District Health Boards are responsible for planning, funding and provision ~33% buy complementary coverage and supplementary coverage	Medicare provides insurance for those aged 65 and older, some disabled; Medicaid provides insurance for low-income; for those without employer coverage, state-level insurance exchanges exist with income-based subsidies ~66% of population is covered by primary private voluntary insurance (employer-based and individual)
Funding for mental health	Mental health-related general practitioner (GP), psychologist and specialist consultations are reimbursed by Medicare; inpatient admissions to public hospitals are free	Mainstream GP, specialist and hospital mental health services are provided free by provinces and territories; National government supports mental health services for a subset of populations	District Health Boards fund community and institutional care for mental health needs; inpatient and outpatient public hospital services are free	Most private health insurance plans are required to cover mental health and substance use disorder services; all Medicaid and Medicare cover some mental health services
Health service system applicable to Indigenous population	Largely administered by mainstream organisations; some care is provided by the National Aboriginal Community Controlled Organisation, a network of independent local health services owned and run by local Aboriginal and Torres Strait Islander communities	Through the First Nations and Inuit Health Branch, the federal government delivers certain mental health services and funds non-insured health benefits (including counselling) to eligible First Nations and Inuit communities	Primary Health Organisations, funded by District Health Boards, are customised to their enrolled populations, sometimes with a focus on the Maori population	The federal government fully funds health services, including mental health services, for Native Americans and Alaska Natives through a combination of Medicaid and care delivered by the Indian Health Service

Sources: [91–94]

The higher Indigenous burden likely results, at least in part, from inadequate treatment, unmet needs, and barriers to receiving appropriate and effective mental healthcare. There is evidence from epidemiological studies that Indigenous people diagnosed with a mental disorder use mental health services at different rates than other ethnic groups [6, 8]. Of Māori people with any mental disorder, only 32.5% visited services for mental health purposes, compared to 41.1% of other ethnic groups [6]. In one Native American tribe, women with any mental disorder made fewer visits for mental health to both specialty (30.8%) and general medical providers (22.6%) than their general population counterparts (33.8% and 42.7%, respectively) [8]. The hospitalisation rate for mental disorders in Australian Indigenous people is about double that of other Australians [11]. This situation is not unique to mental health; Indigenous people have lower overall health service utilisation rates than other ethnic groups and higher hospitalisation rates for any reason [12–16]. Many factors can explain high rates of hospitalisation in a population, including a higher prevalence of illness as well as poorer access to, and availability of, other medical services [11].

Our understanding of the rates and characteristics of mental health service use in the general population has become increasingly sophisticated during the past 30 years. The definition of service use has also been debated and is evolving; with the focus shifting from the types and volume of services received, toward a multi-dimensional approach that encompasses quality of care, person-centeredness, comprehensiveness, integration, continuity, accessibility and cost of healthcare [17]. WHO’s World Mental Health (WMH) survey initiative represents an unprecedented effort to standardise and systematically examine mental health treatment across countries. The WMH survey initiative and other epidemiological surveys have increasingly focused on describing the frequency and volume of evidence-based treatments being delivered, and on identifying unmet needs for care and barriers to care [18]. These advances have enabled the quality of care being delivered to be estimated and individual treatment preferences to be identified, in turn highlighting possible service system deficiencies. For example, by comparing actual treatment delivered with recommendations from evidence-based treatment guidelines, epidemiological survey data has revealed disparities in rates of ‘minimally adequate treatment’ received by different racial or ethnic subpopulations [19–21]. In the United States, one study showed that African Americans with major depressive disorder had significantly lower odds of receiving guideline-concordant treatment for depression than Whites [19]. Latinos with probable mental illness are also less likely than Whites to have received minimally adequate care in the United States [21]. Notably, however, rates of minimally adequate treatment for Indigenous people have not been reported.

Survey design factors account, in part, for limitations in the available information about service use for mental health by Indigenous populations. General population surveys rarely capture a large enough number of Indigenous respondents to draw conclusions about the extent and nature of their service use for mental health [22], although there are exceptions [23]. Even if they did include samples of sufficient size, question content may not acknowledge specialty Indigenous health services and interventions available or relevant for Indigenous people, such as traditional healers and culture-based interventions [22]. While some reports from Indigenous surveys have described aspects of service utilisation, these vary widely in scope and there is limited standardisation of the aspects of service use ascertained. Collectively, these limitations in available information hinder population-level planning for evidence-based service development for Indigenous people.

The aim of this study was to review the service use questions in Indigenous mental health population surveys to determine whether the information needed to guide service development is being collected. First, we asked ‘how many epidemiological surveys have measured service use for mental health among Indigenous populations?’ Second, ‘what elements of service use have these surveys included, and how do they compare with each other and with general population mental health surveys?’ Using this information, we then considered how service use questions in surveys of Indigenous people might best be asked in order to (1) achieve the specificity required for Indigenous contexts and (2) maintain the thoroughness of general population mental health surveys.

## Methods

We conducted a rapid review of surveys that assessed service use for mental health among Indigenous people. It comprised a systematic review of existing syntheses of the literature to identify relevant surveys and focused web searches to identify additional surveys. We considered that a rapid review strategy would identify the relevant surveys given that community representative surveys are large, visible undertakings that would have been published or described in the public domain. Furthermore, the potential use for our study to support the immediate development of future surveys of Indigenous people made a rapid review methodology appropriate [24]. Methods for the rapid review involved three steps, as described below.

### Step 1. Identifying epidemiological surveys measuring service use for mental health in Indigenous populations

#### Search strategies

A systematic review was conducted according to the Preferred Reporting Items for Systematic Reviews and Meta-Analyses (PRISMA) guidelines [25]. We focused on existing syntheses of the literature in which authors identified surveys designed to measure mental health status and/or service use among Indigenous people. Reviews were sought from Australia, New Zealand, Canada and the United States. We searched PubMed (including Medline), PsycINFO and CINAHL with a broad search string that used Boolean logical operators to combine terms specifying population (e.g. Indigenous, ‘First Nations’, aboriginal, Métis, Māori, or ‘American Indian’); condition (e.g. ‘mental disorders’ or ‘mental health’); setting (e.g. ‘healthcare use’ or ‘mental health services’); and information source (e.g. ‘review’). See Additional file 1 for the search strategy.

Because epidemiological surveys are often conducted by governments, the academic search was augmented by web searches to locate relevant grey literature. In particular, we focused the web searches on government department websites of states or provinces with relatively high Indigenous populations, as indicated by census data.

We also searched citation lists of key research papers and reports found in the above searches.

#### Eligibility criteria

We included reviews that described at least one survey meeting all of the following eligibility criteria: drew a national or state-level, community-representative sample of adult (15+ years) Native American, Inuit, Métis, First Nations, Māori, Australian Aboriginal or Torres Strait Islander peoples, or sampled sufficient Indigenous adults in order to obtain reliable estimates for subgroup analyses; collected data during or after 1990 (to ensure relevance to contemporary service systems); and measured the use of interventions or services, or consultations with health professionals, for mental health reasons. Surveys conducted with restricted samples (e.g. samples recruited from healthcare or other service settings; individuals with selected health conditions) were excluded. Surveys of individuals exclusively under age 15 were excluded because of the uniqueness of the service context for this age group. Hereafter, the eligible surveys are referred to as ‘surveys of Indigenous people’.

From the included reviews, we identified all surveys meeting the above criteria. To this, we added further surveys identified through the web searches.

#### Obtaining survey instruments

For each survey meeting the eligibility criteria, we sought access to the interview schedules through web searches and by contacting relevant authors when schedules were not publicly available. Where interview schedules could not be obtained, we relied on published information describing survey content. Only the most recent version of a survey was included, unless relevant aspects of methodology had changed between versions.

### Step 2. Developing a framework to guide review of service use components

We developed a framework to organise the components of service use for mental health included in each of the eligible surveys. The framework was based on inspection of the most recent and comprehensive population mental health survey from each of the four countries of interest [23, 26–28]. Each of these survey instruments was based on the Composite International Diagnostic Interview (CIDI) [23, 27–29], a fully-structured interview designed by WHO for the assessment of mental disorders and treatment received for them [26]. The CIDI-based general population mental health surveys were used as a basis for the review framework because, collectively, they considered a broad range of topics in the assessment of mental health service use [18]. Hereafter, these general population mental health surveys are referred to as ‘general population surveys’. The framework was populated with survey content in relation to individual respondents’ reported use or perceived need for mental health services.

### Step 3. Reviewing service use components

Information was extracted from each Indigenous survey and summarised into templates. We recorded survey descriptors as follows: survey name, design, year(s) of data collection, sample size, sampling strategy, sample characteristics and availability of interview schedule. We then summarised whether and to what degree the survey’s service use content addressed each of the components in our framework. Comparison within and across surveys was narrative, with a focus on identifying common inclusions and omissions, and the inclusion of Indigenous-specific content (e.g. the inclusion of culturally specific practitioners, services, interventions or barriers to care). Data extraction was undertaken by two authors (CM and SL), in consultation with a third (MH) as necessary.

## Results

### Step 1. Identifying epidemiological surveys measuring service use for mental health in Indigenous populations

**Fig. 1:**
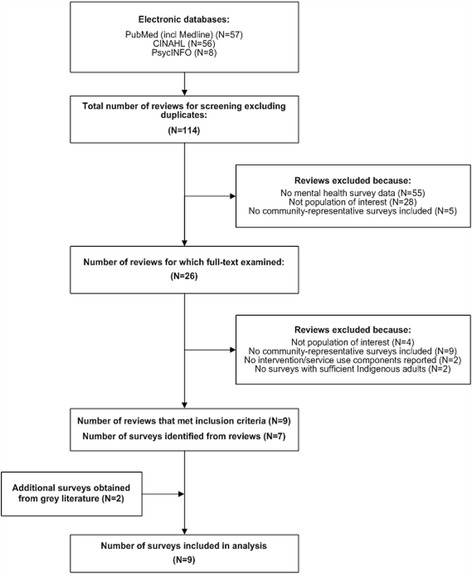
Results of the systematic search

The results of the search strategy are summarised in Fig. 1. Nine surveys met our criteria. Seven of the surveys were identified from the reviews identified in the database search. The topics of those reviews were prevalence reports of mental and substance abuse disorders (*n* = 5), engagement of Indigenous people with health services (*n* = 2), and psychiatric or psychological assessment tools (*n* = 2). A further two surveys were identified via the web searches. These were state health surveys with mental health components that sampled sufficient numbers of Indigenous adults.

The key characteristics of the included surveys are summarised in Table 2. Six surveys were conducted exclusively with Indigenous respondents – four in Canada [30–33], one in Australia [34] and one in the United States [35]. Three further surveys, one from New Zealand [23] and two from the United States [36, 37], were general population surveys that oversampled Māori and American Indian/Alaska Native people, respectively, in order to generate robust estimates for these groups. The number of Indigenous respondents in each survey ranged from approximately 319 to 61,041. Two iterations of the Aboriginal Peoples Survey from Canada were included, one from 2006 [30] and one from 2012 [31], because their content changed sufficiently between versions to justify including both.

**Table 2:** Summary of Indigenous mental health surveys from Australia, New Zealand, Canada and the United States

Study	Year(s) of data collection	Sample size	Sampling strategy	Sample characteristics	Survey Aim(s) and Scope
Aboriginal People’s Survey (Canada) [30]	2006	61,041	Sample was selected from individuals who reported Aboriginal identity, Aboriginal ancestry, registered Indian status, or Indian band membership on the 2006 Census questionnaire; questions were administered in telephone interviews and personal interviews and responses were recorded on paper	The target population was composed of all people living in Canada who have North American Indian, Métis or Inuit identity or ancestry, excluding people living in Indian settlements or on-reserves; adult age range: 15+	The aim of the survey was to identify the needs of Canadian Aboriginal people, including their health needs; other focus areas included language, employment, income, schooling, housing and mobility
Aboriginal People’s Survey (Canada) [31]	2012	~28,410	Sample was selected from individuals who reported Aboriginal identity, Aboriginal ancestry, registered Indian status or Indian band membership in the 2011 National Household Survey; questions were administered in telephone interviews and personal interviews and responses were recorded on a computer	The target population was composed of the Aboriginal identity population of Canada living in private dwellings, excluding people living on Indian reserves and settlements and in certain First Nations communities in Yukon and the Northwest Territories; adult age range: 15+	The aim of the survey was to identify the needs of Canadian Aboriginal people, including their health needs; other focus areas included language, employment, income, schooling, housing and mobility
American Indian Services Utilization, Psychiatric Epidemiology, Risk and Protective Factors Project (AI-SUPERPFP; United States) [35]	1997–1999	3084	Individuals were randomly selected from tribal rolls; trained tribal members interviewed participants face-to-face; data collection was computer assisted	The AI-SUPERPFP was a large-scale, multi-stage, cross-sectional study of the prevalence of DSM disorders and help-seeking behaviour among two of the larger tribes in the United States (Southwest and Northern Plains Indians); age range: 15–54	The authors identified five aims of the survey: 1. To measure the prevalence of major DSM disorders (including culture-specific syndromes) among two Native American tribes 2. To measure service use for mental health, including the use of services provided by the Indian Health Service, other biomedical service providers, and by traditional medicine men and/or healers 3. To examine the relationships between stress, mediators, psychiatric morbidity and predisposing factors 4. Compare the survey’s results with similar data gathered in other studies 5. Gather ethnographic information that will allow cultural contextualisation of the results
Arizona Health Survey (United States) [37]	2010	~319 (number of Native American or American Indian respondents)	Participants were selected by a random digit dial method on landlines; interviews were conducted by telephone and data collection was computer assisted	The survey sample is representative of Arizona’s non-institutionalised population living in households with landline telephones; the sample was geographically stratified to represent Maricopa County and the remainder of Arizona	The aim of the survey was to collect information on the health and health-related behaviours, access to healthcare, and various health-related demographic, social and environmental factors of the Arizona population; regarding service use specifically, the survey aimed to collect information on: • Community strengths, resources, barriers to care and need for care • Attitudes toward prevention and utilisation
Australian Aboriginal and Torres Strait Islander Health Survey (AATSIHS; Australia) [34]	2012–2013	~12,900	Sample was selected using stratified multi-stage sampling from Aboriginal and Torres Strait Islander dwellings identified from 2006 and 2011 Census data; information was collected by face-to-face interviews	The AATSIHS aimed to be a nationally representative survey of Aboriginal and Torres Strait Islander people who were residents of private dwellings in remote and non-remote areas throughout Australia; adult age range: 18+	The aim of the survey was to collect information on the health status and related demographics of Aboriginal and Torres Strait Islander people, including: • The health of the population, including the existence of chronic health conditions • Health risk factors • Use of health services such as consultations with health practitioners and other help-seeking behaviour • Demographic and socioeconomic factors
California Health Interview Survey (Adult) (United States) [36]	2013–2014	574 (number of American Indian or Alaska Native respondents)	Participants were selected by a random digit dial method on cell phones and landlines; interviews were conducted by telephone	Survey data provides population-based estimates for California’s American Indian and Alaska Native population; age range: 18+	The aim of the survey was to collect information on the health status and healthcare access issues of the population of California, including: • Health status, conditions and behaviours, including mental and dental health • Access to and use of health services • Neighbourhood and housing • Food environment • Health insurance • Eligibility for public programmes • Employment and income
The First Nations Regional Health Survey – Adult (Canada) [32]	2002–2003	22,602	First Nations fieldworkers were trained to administer the surveys within their communities, usually in the respondent’s home in face-to-face interviews; data collection was computer assisted	The survey sample was designed to represent the First Nations population living in First Nations communities in all provinces and territories, except Nunavut; overall, 216 communities were included and 5.3% of the target population was surveyed; adult age range: 18+	The aim of the survey was to improve First Nations’ research capacity, and generate health information usable and interpretable from a First Nations’ perspective; the survey was designed to collect information on: • Community needs • Services provided within First Nations communities • Associated factors, underlying causal relationships, motivations for specific behaviours and how changes over time influence health and wellness
New Zealand Mental Health Survey (New Zealand) [23]	2003–2004	2595 (number of respondents of Māori ethnicity)	Households were randomly selected and then an individual within the household was randomly selected; interviews were conducted face-to-face and responses were collected on a computer	The survey design was for a nationally representative sample of people living in permanent private dwellings throughout New Zealand; to improve the precision of estimates for Māori and Pacific people, oversampling was used; age range: 16+	The aims of the survey were to: • Describe the prevalence rates of major mental disorders overall and by social and demographic factors of the New Zealand population • Describe the disability burden associated with mental disorder • Describe and compare patterns of health service use and barriers to care for people with mental disorders, specifically patterns related to ethnicity and sociodemographic correlates
The Nunavik Inuit Health Survey (Canada) [33]	2004	1006	The survey was conducted using a complex two-stage stratified random sampling; the first stage was to select a stratified random sample of private Inuit households with proportional allocation, in the second stage, all eligible people were asked to participate according to the survey steps or instruments; the survey was based on self-administered and interviewer-completed questionnaires	The target population of the survey was permanent residents of Nunavik, excluding residents of collective dwellings and households in which there were no Inuit aged 18 years and over; age range: 15+	The aim of the survey was to collect social and health information on the Canadian Inuit population, including information on various health indicators, physical measurements, and information on social, environmental and living conditions; regarding service use specifically, the survey aimed to collect information on: • Inuit health service use and use of certain medications • Preventative behaviour • Perceptions of health and well-being among the Inuit

The surveys’ aims and scope varied with respect to their relative emphases on the measurement of health or mental health, the measurement of mental disorders or more broadly defined mental health problems, the aspects of service use assessed, the range of health-related characteristics assessed, and whether they were designed to facilitate comparison to other populations. All nine surveys indicate in their methodology reports [23, 32, 33, 35, 37–41] that the survey’s sampling strategy and/or survey content was designed with cooperation or input from Indigenous people. In the information available online, more specific collaboration with Indigenous people was described for five surveys from Canada, New Zealand and the United States [23, 32, 35, 39, 40]. For example, the First Nations Regional Longitudinal Health Survey was developed with Indigenous people and replaced a Western-based analytical framework with one based on First Nations principles. The culturally appropriate interpretation model served as a basis for the survey’s content design and allowed the information to be presented back to Indigenous communities in a way that was usable and reinforced a First Nations’ perspective [32].

### Step 2. A framework to guide review of service use components

We distilled the service use content of the four general population surveys into a framework comprising nine components, namely hospital admissions, medications, support services or self-management strategies, types of health professionals consulted, number, duration, payment and location of health professional consultations, interventions received, perceived need for care, barriers to care, and treatment dropout. Table 3 summarises the components of the framework, and the main questions and/or response options used to assess each component. Each general population survey collected information on eight or more of the nine service use components. Canada’s survey assessed all of the nine components. Within components, there was some variability, for example, the surveys from the United States and New Zealand omitted questions about perceived need for care, and Australia’s survey did not include questions about treatment dropout.

**Table 3:** A framework to guide review of service use components

Component	Summary of content
Hospital admissions	Questions about hospital admissions for mental health asked respondents about: • number of admissions • date of admission • age at time of admission • duration of admission • (medical reason for admission)
Medications	Questions about medications taken or prescribed for mental health asked about: • prescriptions received from a physician • age at time of receipt of prescription or length of time they had been taking the medication • herbal medicines; ‘recommended/prescribed’ herbal medicines • the professional who recommended the herbal medicine • (types of medication taken in the past 2 weeks – up to five types of medication could be recorded) • (number of medications taken) • (whether medications were taken according to the recommended dose)
Health professionals consulted	Most surveys asked which professionals had been consulted in the past 12 months; commonly listed professionals were: • psychiatrist • psychologist • social worker • counsellor • other mental health professional • general practitioner or family doctor • other medical doctor • nurse, occupational therapist or other health professional • religious or spiritual advisor • other healer Some surveys asked about the modality of the consultation, including whether the health professional was: • seen in person • talked to over the phone
Number, duration, payment and location of health professional consultations	Surveys collected specific information about the respondents’ consultations with health professionals, asking for: • age at first and last visit • frequency and duration of visits • (how many different doctors or clinics were visited) • location of visits • total money spent on mental health treatment in the past 12 months out-of-pocket • payment method
Interventions received	Survey questions asked about types of interventions received, options included: • telephone psychic or telephone counsellor, including duration and topic of call • counselling • alternative therapies (e.g. acupuncture, biofeedback, hypnosis, massage therapy, etc.) • (psychotherapy) • (cognitive behavioural therapy) • (help to sort out housing or money problems) • (help to improve your ability to work, or to use your time in other ways) • (help to improve your ability to look after yourself or your home) • (help to meet people for support or company)
Support services or self-management strategies	Several different support services or self-management strategies were listed as options, including: • the internet for information • support group or chat room • self-help group • hotline or telephone counselling service • psychological counselling or therapy • (self-coping strategies) • (services provided by employer)
Perceived need for care	Surveys asked respondents: • whether or not they felt they received as much help as they needed for problems related to mental health in the past 12 months • what specific treatments they felt that they did not receive, e.g. not enough medicine or tablets or not enough talk therapy
Barriers to mental healthcare	Surveys inquired about barriers to care. Themes included: • financial barriers • self-reliance • knowledge and beliefs about treatment • stigma from others and discrimination • practical barriers and availability • (spirituality and faith)
Treatment dropout	Surveys also asked participants if they completed the recommended course of treatment and barriers to continuing care; listed reasons for dropout included: • self-reliance • stigma • beliefs about treatment • practical reasons and cost

Sources: [1, 23, 27, 42]

Parentheses indicate content specific to one survey; all other content was present in at least two surveys

### Step 3. Review of service use components in surveys of Indigenous people

We summarised whether the surveys of Indigenous people’ service use content addressed each of the components in our framework (Table 4). A more detailed narrative comparison across surveys is presented below.

**Table 4:** Service use components included in epidemiological surveys of Indigenous people

Survey	Interview schedule obtained	Hospital admissions	Medication	Health professionals consulted	Number, duration, payment and location of consultations	Interventions received	Support services or self-management strategies	Perceived need for care	Barriers to care	Treatment dropout
Aboriginal People’s Survey 2006 (Canada) [30]	✓		✓	✓	✓		✓	✓	✓	
Aboriginal People’s Survey 2012 (Canada) [31]				✓				✓	✓	
American Indian Services Utilization, Psychiatric Epidemiology, Risk and Protective Factors Project (United States) [35]	✓	✓	✓	✓	✓	✓	✓	✓	✓	
Arizona Health Survey (United States) [37]	✓		✓		✓			✓	✓	
Australian Aboriginal and Torres Strait Islander Health Survey (Australia) [34]	✓			✓	✓	✓		✓	✓	
California Health Interview Survey – Adult (United States) [36]	✓		✓	✓	✓			✓	✓	
First Nations Regional Health Survey – Adult (Canada) [32]	✓		✓	✓			✓		✓	
New Zealand Mental Health Survey (New Zealand)	✓	✓	✓	✓	✓	✓	✓	✓	✓	
The Nunavik Inuit Health Survey (Canada) [33]	✓		✓	✓			✓			
N (out of 9):	9	2	7	8	6	3	5	7	8	0

A ✓indicates that the component was present in the survey

Two of the nine surveys of Indigenous people (New Zealand and United States) asked about hospitalisation for mental health or substance abuse reasons [23, 35]. Both of the surveys included questions about admission to Indigenous-specific hospitals (a hospital-based Maori mental health service and an Indian Health Service hospital). Only one survey was as comprehensive as the general population surveys, asking about the number of hospital admissions in the respondent’s lifetime and in the past 12 months, when those admissions happened, the respondent’s age at time of admission and the duration of admission [23]. The other survey only asked about the number of overnight admissions and outpatient services received in the past year [35]. While this survey was less thorough, the questions did differentiate between admissions for mental health reasons and for alcohol- or drug-related reasons, unlike all but one of the general population surveys [42].

Five surveys of Indigenous people (Canada, New Zealand and United States) included questions about the use of support services or self-management strategies such as telephone counselling, support groups, instrumental help from family or friends and spiritual practices. Collectively, the content of the surveys of Indigenous people largely covered that of the general population surveys, but no individual Indigenous survey was comprehensive. One survey asked about the use of telephone counselling [32]. Two surveys of Indigenous people asked about participation in support groups, such as various 12-step programmes like Alcoholics Anonymous [23, 35]. Four surveys of Indigenous people incorporated questions on spirituality or religion, asking about reliance on spiritual practices for emotional guidance [23, 30, 33, 35]. Notable omissions from surveys of Indigenous people were questions about self-coping strategies and internet use related to mental health or substance use help-seeking.

Seven surveys (Canada, New Zealand and United States) asked about medication taken to treat mental disorders [23, 30, 32, 33, 35–37]. The amount of detail collected in the surveys was highly variable. Only one of the surveys’ content was as comprehensive as the general population surveys, including a large list of names of psychiatric medications (i.e. brand names) and classes of medications, questions about which medical professional prescribed the medication and any use of non-prescription drugs. Two surveys asked about specific classes of medication and included the names of traditional Indigenous medicines [23, 35]. One survey asked about any prescription medications taken almost daily for 2 weeks or more for an emotional or personal problem in the past 12 months [36]. No survey assessed the duration of medication use or medication adherence.

Eight out of the nine surveys of Indigenous people (Australia, Canada, New Zealand and United States) asked about health professionals consulted for mental health. Five surveys included as diverse a range of professionals as the general population surveys, asking about mental health specialists, other medical professionals and non-medical consultants such as religious figures [23, 30–32, 35]. Six surveys included Indigenous-specific response options, such as healers, roadmen, medicine men, tohungas or Māori healers [23, 30, 32–35]. One survey asked whether the respondent had seen a health professional of the same ethnicity [23] and two asked whether the respondent would prefer to see an Indigenous professional or a doctor from an Indigenous health service [34, 35]. The surveys of Indigenous people did not ask about the modality of the consultation with the health professional (e.g. face-to-face, telephone or online).

Six surveys (Australia, Canada, New Zealand and United States) asked about the number, duration, payment and location of health professional consultations for mental health reasons [23, 30, 34–37]. Three surveys asked about visits to specific locations such as community health centres, doctors’ offices, an Indian Health Service hospital or residential treatment [30, 34, 35]. One survey asked about the number of visits for mental health reasons in the last 4 weeks [35] and one asked about the number of visits in the past 12 months [36]. Regarding payment, two surveys from the United States asked if the respondent’s insurance covered treatment for mental health problems [36, 37] and one from Australia asked whether or not the respondent was covered by private health insurance, reasons for purchasing private health insurance and the extent of the insurance’s coverage, although in this case the questions were not specific to mental healthcare [34].

Interventions received were not consistently assessed. Three surveys of Indigenous people (Australia, New Zealand and United States) asked about interventions received, with a focus on counselling sessions [23, 34, 35]. One survey of Indigenous people asked about alcohol treatment programmes [35] and one asked about substance use education [35]. Other Indigenous-tailored questions included those about use of traditional medicine and wellness practices like tribal ceremonies [23, 35]. No surveys of Indigenous people included questions addressing the use of other forms of therapy such as psychotherapy, cognitive-behavioural therapy or alternative therapies. No surveys of Indigenous people included questions about the use of web-based interventions for mental health and substance use treatment; however, these did not feature in general population surveys, most likely because they were conducted before most developments in this area occurred.

Eight surveys (Australia, Canada, New Zealand and United States) assessed perceived need for care [23, 30–32, 34–37]. Questions around unmet need asked if there was ever a time, and if there was a time in the past 12 months, that the respondent felt they needed care but did not receive it. Unlike the general population surveys, however, surveys of Indigenous people did not assess partially met needs or ask about what specific mental health services the respondent felt they needed but did not receive.

Barriers to care were assessed in eight of the nine surveys (Australia, Canada, New Zealand and United States). The barriers to care content was generally as comprehensive in surveys of Indigenous people as in the general population surveys, but different barriers had greater prominence in the surveys of Indigenous people. Communication problems (e.g. language problems or difficulty understanding each other) were listed as an option in five surveys of Indigenous people [30, 31, 34, 35, 37] as a barrier to seeking care, while only one general population mental health survey included this option. The practical factor listed most frequently in surveys of Indigenous people was not having a service available in the local area [30–32, 34, 35, 37]. Other unique barriers listed included the service not being culturally appropriate [32, 34] and a dislike of doctors [30, 31, 34, 35].

No surveys included questions about treatment dropout.

Questions or response options tailored for the Indigenous context included questions around spirituality, response options for Indigenous health professionals and traditional healers, response options for traditional health and wellness practices, alcohol treatment programmes and substance use education programmes, and response options for communication barriers, the service not being culturally appropriate and a dislike of doctors. Notable omissions from surveys of Indigenous people included questions around treatment dropout, response options for perceived need for care that include type and degree of perceived need, response options for interventions received that included web-based interventions, and support services response options that include web-based information sources. Excluding the omission of web-based content, then, the main gaps in the Indigenous survey content correlate with the updates to the most recent WMH surveys that have introduced these questions. Health service system differences (Table 1) were reflected in questions about Indigenous-specific hospital admissions and questions about payment with insurance. The survey from the United States and the survey from New Zealand covered content from eight of the service use components, the three surveys from Canada covered from three to six components, and the survey from Australia covered five components.

## Discussion

In summary, surveys of Indigenous people commonly assessed medication, health professionals consulted, number, duration, payment and location of consultations, barriers to care, and perceived need for care. Certain components of mental health service use were commonly tailored for Indigenous people (types of support services or self-management, types of health professionals consulted, barriers to care and interventions received). Other components were absent or abbreviated, when compared with the most up to date general population surveys (treatment dropout, response options regarding type and degree of perceived need for care, and the use of web-based interventions). Questions regarding medication, interventions received, and number, duration, payment and locations of health professional consultation were the most inconsistent in content between surveys of Indigenous people. Together, these findings suggest some areas for potential focus in the design of future surveys of Indigenous people in Australia, Canada, New Zealand and the United States.

That said, the variability between the surveys of Indigenous people may, in part, reflect their varying aims and scope. For example, those designed to enable comparability with other populations tended to include a large range of mainstream intervention types and health professionals consulted [23, 35]. One other, designed for use in a discrete Indigenous community, included questions about perceived barriers to care due to discrimination [32]. Some surveys considered health more broadly than just mental health, and did not measure service use for mental health independently from physical health service use. This makes disaggregating the former difficult [30, 31]. In contrast, surveys designed to focus on mental health specifically [23, 35] tended to include more detailed questions within and across the components of the framework.

### Strengths and limitations

Several limitations of this rapid review deserve attention. First, our review focused on survey content relating to individual respondents’ use or perceived need for mental health services. We recognise, however, that the surveys assessed other factors that may influence individuals’ help-seeking. Notably, for example, three surveys of Indigenous people captured respondents’ general perceptions of the availability of mental healthcare in the community, including culturally appropriate mental health services. These questions were asked of all respondents, regardless of whether they had used or wanted mental healthcare. It was beyond the scope of this review to examine such factors, but these could be the focus of a future review. Second, we only looked at community representative surveys, and it might be that there is thorough service use content in other studies specific to particular clinical, service or community contexts. Third, we are aware that there is significant between- and within-group diversity among Indigenous populations in these countries, but because the surveys we examined do not generally distinguish between different Indigenous subgroups, we have reported on them collectively. Fourth, because our study focused on surveys of Indigenous populations from high-income countries, our findings may not apply to lower or middle-income countries with less well-developed mental health service systems. Finally, it was beyond the scope of this study to consider other aspects of survey design, such as sampling methods or the cultural challenges inherent to using surveys as an assessment tool among Indigenous communities [43]. Further work to develop the most Indigenous-appropriate approach to assessment is needed [43].

### Implications

It is useful to consider the findings of this review in the context of available evidence regarding the use and efficacy of different mental health services and interventions for Indigenous people. This may provide guidance regarding the array of services and interventions for which utilisation should be assessed, particularly those that are evidence-based and may thus allow an assessment of the quality of care received. It also provides guidance as to whether the questions assessing service use for mental health in general population surveys should be modified for use in surveys of Indigenous people. That said, the extent to which particular aspects of service use, or response options, may apply to the design of future surveys will depend on the aim and scope of those surveys, feasibility and the culture- and nation state-specific contexts of service delivery.

#### Hospital admissions

Data from Australia and the United States indicate that Indigenous people are hospitalised or seen in the Emergency Room for mental health reasons at higher rates than non-Indigenous people [44, 45]. However, there has been little survey-based data published on hospital admissions for mental health reasons among Indigenous people, or hospitalisations for physical health problems among Indigenous people with mental disorders. Information on the relative rates of hospitalisation within a population, particularly the rates of avoidable hospitalisations, can help identify unmet or partially unmet needs, and whether or not health services are being used efficiently [16]. Where this is of interest, future surveys of Indigenous people might ask respondents about the number of hospitalisations, admission to Indigenous-specific hospitals (in countries where they exist), date of admission, age at time of admission, duration of admission, and medical reasons for admission; ideally, these would be recorded separately for mental and physical health reasons.

#### Support services or self-management strategies

Research suggests that the church and traditional healers can be the most common sources of emotional help for Indigenous people [46], with spirituality strongly related to Indigenous understanding and management of mental health and substance use issues [47, 48]. Few studies have systematically assessed the effectiveness of spiritual practices, with the exception of some research that has reported on the use and possible efficacy of plant-based ceremonial medicines for treating substance abuse [49, 50]. Although there is a limited evidence base for its effectiveness at this time, spiritual practices are culturally important. Asking an individual about avenues of support received from their spirituality for mental health concerns will inform an understanding of the dialogue between Indigenous constructs of wellness and Western treatment methods for mental health problems [47].

Support groups, particularly 12-step programmes such as Alcoholics Anonymous, are reported to be popular in Indigenous populations for alcohol and drug abuse treatment [51, 52], although little is known about their benefits [51]. Given the widespread use of these programmes, surveys of Indigenous people could model questions about support groups for alcohol use, substance use and emotional reasons from the general population surveys.

Recent studies have indicated that the internet is an effective platform for reaching Indigenous populations with health information and resources [53–55]. The minimal availability of culturally appropriate health information online has been noted in the literature [53]; however, neither the use of culturally-specific websites, nor their impact, has been systematically assessed. Surveys of Indigenous people could include questions about online information retrieval and online support group participation, including the use of culturally specific resources, such as the ‘yarning places’ message boards on Australian Indigenous HealthInfoNet [56] or the Native Health Database [57].

#### Medication

Pharmacy data, observational study data and randomised control trials of Indigenous people suggest that Indigenous people are prescribed and tolerate medications for mental disorders at similar rates to non-Indigenous people [58–62]. The pharmacoepidemiology sections of the CIDI ask about the use of prescription and non-prescription medications (usually in the past 12 months) for mental health and substance use reasons, professional supervision, duration of use and adherence [18]. A list of prescription medications is provided as a visual aid [18]. Together, this data can provide information about the extent to which people are receiving appropriate pharmacological treatment.

While not widely documented, there is evidence that some Indigenous people view traditional medicine and biologically based therapies as an important element of mental health treatment [63, 64]; the inclusion of questions about the use of traditional and plant-based medicines would allow this information to be captured.

#### Health professionals consulted

There is evidence that Indigenous people are more likely to seek help from traditional healers than from other medical providers, including mental health specialists, and report greater satisfaction with care provided by a traditional healer compared with mental health specialists [6, 32, 65]. Some Indigenous people report using both traditional and allopathic methods to treat emotional or substance abuse problems [66].

The availability of mental health staff who share a culture or ethnicity may be a factor determining mental health service use for Indigenous people [46, 67, 68], but is seldom captured by survey data. The need for Indigenous representation in the workforce has been reflected in a range of services [69, 70]. In New Zealand, publicly provided healthcare is often customised to treat the local Maori population, including the hiring of Indigenous health workers. Unlike the other surveys of Indigenous people, the New Zealand Mental Health Survey asks if the respondent had ever seen a Maori mental health specialist. Information about the use of traditional healers and the perceived efficacy of treatment by Indigenous health professionals may also warrant capture.

#### Number, duration, payment and location of health professional consultations

There is limited data on the frequency of healthcare visits among Indigenous people. When combined with evidence-based guidelines for minimally adequate treatment, information about intervention types, professional consulted, frequency of visits and duration of visits can be used to measure treatment adequacy [1, 71, 72]. Information about professionals seen, interventions received and location of visit or method of payment can be combined to examine rates of consultation across different sectors or programmes within the mental healthcare system, and to monitor the impact of policy reforms on Indigenous populations [73–75]. As reflected in the existing question content around payment, service system differences (Table 1) will determine the relevant information to be collected about insurance plans and payment.

#### Interventions received

Interventions such as psychotherapy, counselling, motivational therapy and cognitive behavioural therapy have had some success among Indigenous populations with mental or substance use disorders [76]. The effectiveness and availability of culturally specific mental health or substance use interventions among Indigenous people, such as sweat lodge ceremonies and drumming, has also been documented [77–80]. Depending on their aim, future surveys focusing on Indigenous people may need to consider including questions about mainstream interventions as well as questions about culture-based interventions.

Mental health and alcohol abuse interventions for Indigenous people have been delivered successfully online and over the phone [81, 82]. Furthermore, review papers and qualitative analyses from Australia, Canada and the United States have highlighted the relevance of telehealth to rural and remote Indigenous communities and its potential to improve access to mental healthcare [83–86]. Questions that assess the mode of delivery of interventions (e.g. online, by telephone and videoconference) might warrant inclusion in surveys of Indigenous people.

#### Perceived need for care

Perceived need for care is a crucial factor in peoples’ decisions about whether to seek mental healthcare. While some estimates of rates of perceived need for care among Indigenous people are available [11], no studies to date have examined the nature of unmet mental healthcare needs for Indigenous people to the extent they have for the general population. Information about the degree and types of perceived need for care among Indigenous communities will fill a gap in knowledge about the extent of unmet demand for services, the extent to which services are fulfilling consumers’ needs, and treatment preferences among Indigenous people.

#### Barriers to care

Obstacles to service utilisation are likely to be specific for Indigenous people because of dynamic social and cultural processes [65, 87]. Three reports that used data from epidemiological surveys of Indigenous people and one qualitative study identified cultural and communication barriers, perceptions of discrimination, stigma of mental illness and the use of unprofessional sources of care, transport and distance, long waiting times, cost and dislike of services, and lack of Indigenous staff, as common barriers to care among Indigenous people [11, 65, 68, 88]. Studies aimed toward understanding barriers to care among Indigenous people may need tailored response options.

#### Treatment dropout

Once treatment is initiated, there is evidence that racial and ethnic minorities discontinue treatment at rates higher than their White counterparts [89]. While treatment dropout is common, and contributes to poor outcomes and an inefficient use of resources, little is known about its patterns and predictors, which has led to its recent addition to the WMH surveys [90]. The addition of questions about discontinuing treatment will provide important information for assessing barriers to mental health treatment [18] for Indigenous people.

## Conclusions

In order to identify and understand mental health service system deficiencies, there is an urgent need to collect information about Indigenous people’s use of health services that takes into account their specific service preferences and service contexts. This review provides recommendations about changes to future Indigenous survey service use questions to better align their content with the information needed to inform health service planning, and consequently to assist in closing the mental health gap for Indigenous people.

## Additional file

Additional file 1: Search strategy. (DOCX 20 kb)

## Abbreviations

CIDI: Composite International Diagnostic Interview
WMH: World Mental Health.
