# Supplementary material for: Assessing service use for mental health by Indigenous populations in Australia, Canada, New Zealand and the United States of America: a rapid review of population surveys
Source: Health Res Policy Syst. 2017 Aug 4;15:67. doi: 10.1186/s12961-017-0233-5 (PMC5544983; doi:10.1186/s12961-017-0233-5)
Supplement: Additional file 1: — Search strategy. (DOCX 20 kb) [file 12961_2017_233_MOESM1_ESM.docx]

**Additional file 1. Search strategy used in the database search**

**PubMed (Including Medline) search terms**

| **Search** | **Query** |
| --- | --- |
| #1 Population of interest | (((((((((maori[Title/Abstract]) OR aboriginal[Title/Abstract]) OR "american indian"[Title/Abstract]) OR eskimo*[Title/Abstract]) OR "indigenous"[Title/Abstract]) OR "first nations"[Title/Abstract])) OR (((( "American Native Continental Ancestry Group/epidemiology"[Mesh] OR "American Native Continental Ancestry Group/psychology"[Mesh] OR "American Native Continental Ancestry Group/statistics and numerical data"[Mesh] )) OR ("Oceanic Ancestry Group/epidemiology"[Mesh] OR "Oceanic Ancestry Group/psychology"[Mesh] OR "Oceanic Ancestry Group/statistics and numerical data"[Mesh] )) OR ( "Inuits/epidemiology"[Mesh] OR "Inuits/psychology"[Mesh] OR "Inuits/statistics and numerical data"[Mesh] )))) |
| #2 Condition | ((“mental disorders”[MeSH]) OR “mental health”[Title/Abstract]) OR psychiatric[Title/Abstract] |
| #3 Setting | (((("Epidemiology"[Mesh]) OR "Prevalence"[Mesh]) OR “Prevalence”[Title/abstract]) OR “service use”[Title/Abstract]) OR “health care use”[Title/Abstract]) OR “mental health services”[title/abstract]) |
| #4 Information source | ("Review" [Publication Type]) OR "Review Literature as Topic"[Mesh] |
|  | #1 AND #2 AND #3 AND #4 |
| Limits | Publication date from 01/01/1990-20/06/2016; English language; human |

**PsycINFO search terms**

| **Search** | **Query** |
| --- | --- |
| #1 Population of interest | **Index Terms**: alaska natives *OR* **Index Terms**: american Indians *OR* **Index Terms**: indigenous populations *OR* **Index Terms**: inuit *OR* **Keywords**: maori *OR* **Keywords**: first nations *OR* **Keywords**: aborig* *OR* **Keywords**: native american* *OR* **Keywords**: metis |
| #2 Condition | **Keywords**: mental disorders *OR* **Keywords**: psychiatric *OR* **Title**: psychiatric *OR* **Title**: mental health |
| #3 Setting | **Keywords**: health care use *OR* **Keywords**: service use *OR* **Keywords**: mental health services *OR* **Keywords**: prevalence |
| #4 Information source | **Methodology**: Literature Review *OR* Systematic Review *OR* Meta Analysis |
|  | #1 AND #2 AND #3 AND #4 |
| Limits | Publication date from 01/01/1990-20/06/2016; English language |

**CINAHL search terms**

| **Search** | **Query** |
| --- | --- |
| S1 Population of interest | (MH "Aborigines+") OR (MH "Indigenous Health") OR (MH "Native Americans") OR (MH "Maori") OR (MH "Indigenous Peoples+") OR (MH "Eskimos") OR (SU indigenous) OR (SU inuit*) OR (SU first nation*) OR (SU aboriginal) OR (SU metis) OR (SU “american native”) OR (SU maori) |
| S2 Condition | (MH "Mental Disorders+") OR (MH "Substance Use Disorders+") OR (SU “mental illness”) OR (SU “psychiatric disorders”) OR (SU “alcohol use disorder”) OR (SU “drug use disorder”)  OR (SU “mental health”) |
| S3 Setting | (MH "Prevalence") OR (MH "Surveys") OR (MH "Epidemiology+")  OR (SU prevalence) OR (SU epidemiology) OR (SU “health services”) OR (SU “health care use”) OR (SU “health care utilization”) |
| S4 Information source | MW Review |
|  | S1 AND S2 AND S3 AND S4 |
| Limits | Publication date from 01/01/1990-22/06/2016; English language; human |
